# Severe hypoglycaemia and absolute risk of cause-specific mortality in individuals with type 2 diabetes: a UK primary care observational study

**DOI:** 10.1007/s00125-020-05223-3

**Published:** 2020-07-30

**Authors:** Francesco Zaccardi, Suping Ling, Claire Lawson, Melanie J. Davies, Kamlesh Khunti

**Affiliations:** 1 Diabetes Research Centre, University of Leicester, Leicester General Hospital, Gwendolen Rd, Leicester, LE5 4PW UK; 2 Leicester Real World Evidence Unit, Diabetes Research Centre, University of Leicester, Leicester General Hospital, Gwendolen Rd, Leicester, LE5 4PW UK; 3 National Institute for Health Research, Biomedical Research Centre, University of Leicester, Leicester, UK

**Keywords:** Absolute risk, Cardiovascular disease, Causality, Competing risk, Electronic health records, Hypoglycaemia, Mortality, Observational study, Prognosis, Type 2 diabetes

## Abstract

**Aims/hypothesis:**

Several pathophysiological mechanisms would suggest a causal link between hypoglycaemia and cardiovascular death; conversely, current knowledge would not support a causal relationship with other causes of death. To clarify the nature and the magnitude of the association between hypoglycaemia and death, we investigated the 5 year mortality risks for cardiovascular disease, cancer and other causes in individuals with type 2 diabetes admitted to hospital for a severe hypoglycaemic episode.

**Methods:**

We defined in the UK Clinical Practice Research Datalink database a prevalent cohort of adults with type 2 diabetes diagnosed between 1 January 1998 and 1 January 2011 (index date), with available linkage to the Office for National Statistics (ONS) and the Hospital Episode Statistics (HES). A hospital admission reporting hypoglycaemia as the underlying cause was identified before the index date in the HES; date and underlying cause of death were obtained from the ONS. We quantified the 5 year risk of cause-specific death in people with and without admission to hospital for severe hypoglycaemia, adjusting for potential confounders and accounting for competing risk.

**Results:**

Of the 74,610 subjects included in the cohort, 388 (0.5%) were admitted at least once for a severe hypoglycaemic episode; subjects admitted were older, with higher HbA_1c_ and a greater prevalence of multimorbidity. During a median follow-up of 7.1 years, 236 (60.8%) and 18,539 (25.0%) deaths occurred in subjects with and without a previous severe hypoglycaemia, respectively. Non-cardiovascular causes accounted for 71% of all deaths in both subjects with and without hypoglycaemia. In a 60-year-old person with severe hypoglycaemia, the 5 year absolute risk of death, adjusted for age, sex, ethnicity, systolic blood pressure, total cholesterol, HbA_1c_, BMI, eGFR, smoking status, alcohol consumption and deprivation (Townsend score), was 6.6%, 1.1% and 13.1% for cardiovascular, cancer and other causes, respectively, while the 5 year absolute risk difference compared with a subject without severe hypoglycaemia was 4.7% (95% CI 1.0, 8.3) for cardiovascular, −1.4% (−4.1, 1.4) for cancer and 11.1% (6.1, 16.1) for other causes of death. Results were consistent in models further adjusted for medications and comorbidities (myocardial infarction, stroke, peripheral artery disease, heart failure, atrial fibrillation, cancer), with sulfonylurea and insulin associated with increased mortality rates (from cause-specific hazard ratio of 1.06 [95% CI 0.99, 1.14] for cancer death with use of sulfonylurea to 1.42 [1.29, 1.56] for cardiovascular death with use of insulin). Results were robust to missing data.

**Conclusions/interpretation:**

The results of this study indicate severe hypoglycaemia as a marker of, rather than causally linked to, an increased risk of long-term mortality. Regardless of the nature of the association, a severe hypoglycaemic episode represents a strong negative prognostic factor in patients with type 2 diabetes.

**Figure:**
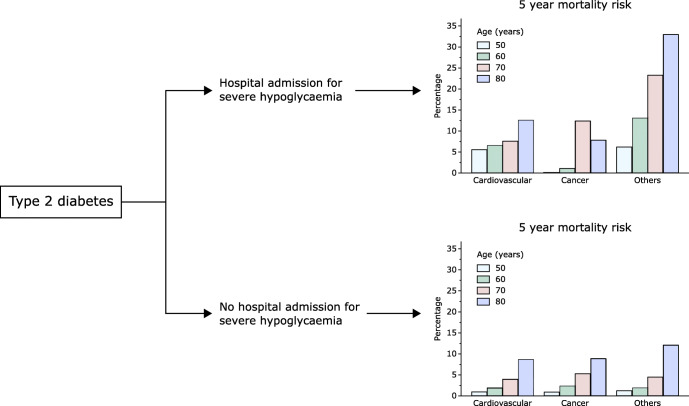
Graphical abstract

**Electronic supplementary material:**

The online version of this article (10.1007/s00125-020-05223-3) contains peer-reviewed but unedited supplementary material, which is available to authorised users.

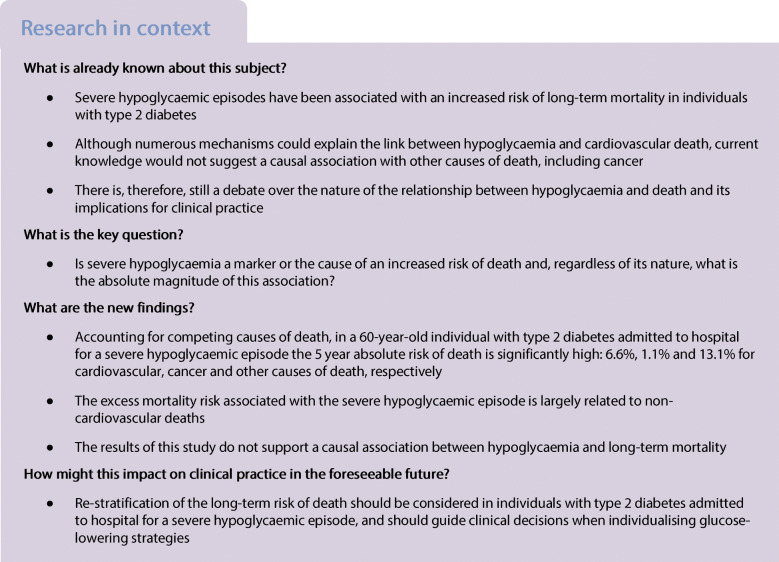



## Introduction

One of the proposed hypotheses to explain the results of the Action to Control Cardiovascular Risk in Diabetes (ACCORD) trial, which showed a 22% higher relative risk of death in individuals with type 2 diabetes randomised to intensive glucose control, was the higher rate of hypoglycaemic events in participants intensively treated compared to those randomised to conventional glucose control [1]. Hypoglycaemia is, in fact, the most common side effect of intensive glucose reduction and, when severe, is potentially associated with fatal complications including injury, arrhythmias, seizures and coma [2]. Such short-term events are well known to clinicians, and experimental studies over the last decades have highlighted the mechanisms linking hypoglycaemia to cardiac, vascular and neurological complications [2].

Whether severe hypoglycaemia could also be associated with long-term complications, however, is unknown. Following the ACCORD publication in 2008, this hypothesis has been variably confirmed or refuted in post hoc investigations of trials as well as in epidemiological studies using cohorts or electronic health records. In the ACCORD study itself, the rate of symptomatic hypoglycaemic episodes requiring medical or non-medical assistance was higher in intensively treated participants [3]; these episodes were also associated with a higher risk of death in both the intensive and the conventional treatment arm [3, 4]. Yet, the risk of death associated with severe hypoglycaemia requiring medical assistance was lower in participants randomised to the intensive control compared to those receiving the conventional treatment [3]. Similar findings were reported in the post hoc analysis of the Outcome Reduction with Initial Glargine Intervention (ORIGIN) trial, for the composite outcome of fatal and non-fatal CVD [5]. Furthermore, a post hoc analysis of the Action in Diabetes and Vascular Disease: Preterax and Diamicron Controlled Evaluation (ADVANCE) trial indicated that participants with type 2 diabetes reporting hypoglycaemic episodes requiring assistance also had an increased risk of cancer and skin, respiratory or digestive diseases [6]. These analyses would suggest severe hypoglycaemia as a marker of other medical conditions that are causally associated with a higher risk of CVD and death. On the other hand, numerous observational studies have also shown an increased CVD and non-CVD risk after controlling for multiple potential confounders [2].

The nature of the association between hypoglycaemia and the long-term risk of CVD and non-CVD death remains unclear, therefore [7]. Notably, the majority of previous investigations reported only the relative risk of individual events and did not account for their competing nature [2]; in fact, a death due to CVD precludes the occurrence of death due to cancer. Recognising the competing risk and estimating the absolute risk is particularly important when investigating the relationship between hypoglycaemia and cause-specific death, for two main reasons. First, as the mechanisms linking hypoglycaemia to death have been mainly postulated for CVD [8], quantifying the risk of CVD death accounting for other causes of death is essential to judge the causality criterion of biological plausibility. Second, irrespective of the nature of the association, estimating the absolute risk gives more insights into the individual and public health burdens associated with hypoglycaemia.

In this context, we designed a retrospective observational study to quantify the absolute risk of death attributable to CVD, cancer and other causes over a period of 5 years in people who experienced a severe hypoglycaemia, defined as an episode resulting in hospital admission.

## Methods

### Data

The Clinical Practice Research Datalink (CPRD) is an electronic primary care database including anonymised longitudinal records on demographic, clinical, laboratory and medication records from about 11 million patients of 674 general practice surgeries in the UK; CPRD patients are largely representative of the national population in terms of age, sex and ethnicity [9]. For this study, we used CPRD patients with available linkage to the Hospital Episode Statistics (HES) database and the Office for National Statistics (ONS). This study was conducted following a pre-specified protocol approved by an Independent Scientific Advisory Committee (CPRD study protocol 18_157R2). All codes used to identify the population, main exposure, covariates and outcomes are available on GitHub (frazac82).

### Cohort definition

**Fig. 1:**
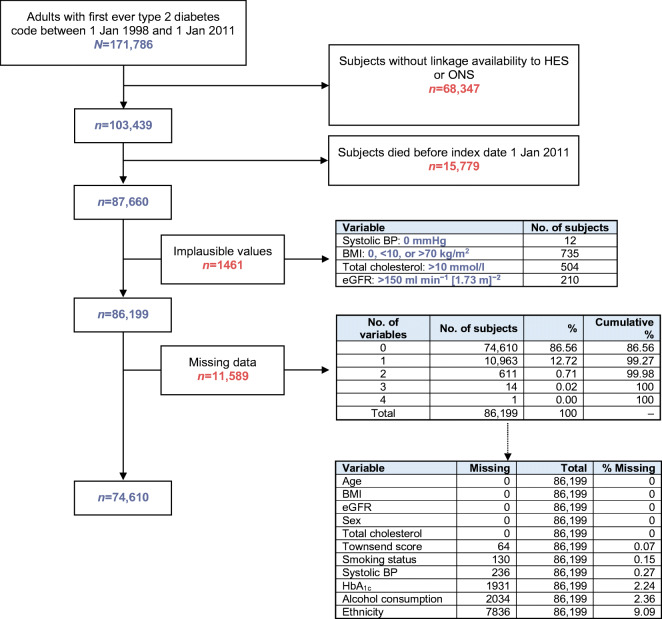
Cohort definition

This observational, retrospective, prevalent cohort study comprised adults aged 18 years or over with a first ever code of type 2 diabetes between 1 January 1998 and 1 January 2011 (index date). From this cohort, we excluded subjects without linkage to HES and ONS and those who died before the index date (Fig. 1).

### Main exposure and covariates

The main exposure was severe hypoglycaemia, defined in this study as an episode of hypoglycaemia resulting in hospital admission. Episodes were identified before the index date in the HES database using International Classification of Diseases (ICD)-10 code E16.0, E16.1 or E16.2 in the first position in the hospital admission record; subjects were considered exposed if they experienced at least one admission to hospital for hypoglycaemia. Other covariates assessed in the CPRD and HES databases were: any time before the index date for medical history of myocardial infarction, stroke, peripheral artery disease, heart failure, atrial fibrillation or cancer; within 6 months before the index date for statin and antiplatelet medications and within 3 months for glucose-lowering medications; at the closest date before the index date for HbA_1c_; and at the closest date before or after the index date for ethnicity, smoking status, alcohol use, systolic blood pressure, total cholesterol, BMI and eGFR. Deprivation was assessed with the 2001 Townsend score.

### Outcomes

Date and cause of death were obtained from ONS death registration data. We used the ICD-10 codes reported for the underlying cause of death in death certificates to define whether mortality was related to cardiovascular, cancer or other causes.

### Statistical analysis

We report the characteristics of participants at index date, stratified by exposure status, as median and interquartile range for continuous data and number and percentage for categorical data.

The analysis quantified the 5 year risk of cause-specific death using the Royston–Parmar–Lambert parametric survival model [10]: the time scale was time into the study, from index date to death or censoring on 14 February 2018 (last ONS linkage update for this study). To adjust for imbalance of covariates at index date and account for competing risk, individual 5 year survival probabilities and their differences comparing exposure (severe hypoglycaemia) vs non-exposure (no severe hypoglycaemia) were estimated and averaged across individuals using the Stata *stpm2* and *standsurv* commands [10]. Complete-case survival regression for the main analysis included the covariates age, sex, ethnicity, systolic blood pressure, total cholesterol, HbA_1c_, BMI, eGFR, smoking status, alcohol consumption, Townsend score and a non-linear interaction between a restricted cubic spline transformation of age (with knots at 33th and 66th centiles of distribution) and the main exposure (model 1); the interaction allowed the effect of hypoglycaemia on death to differ across age. In a sensitivity analysis, we added time since last severe hypoglycaemia to model 1. In model 2, we performed complete-case regressions upon further inclusion of the covariates glucose-lowering medications (metformin, sulfonylurea, insulin, others), comorbidities (myocardial infarction, stroke, peripheral artery disease, heart failure, atrial fibrillation, cancer) and cardioprotective medications (statin and antiplatelet drugs). Lastly, the robustness of the results was assessed after multiple imputation of missing values for variables included in model 2, with estimates across 50 imputations combined using Rubin’s rule. Analyses were performed with Stata IC (StataCorp. 2017. Stata Statistical Software: Release 15.1 College Station, TX: StataCorp) and results are reported with 95% CI; the complete statistical code is available on GitHub (frazac82).

## Results

### Cohort definition and subject characteristics

Of the 87,660 subjects with type 2 diabetes included in the cohort, 1461 had implausible values of covariates and 11,589 had missing data for at least one covariate, leaving a final cohort of 74,610 subjects with complete data for the main analysis (Fig. 1). Of these subjects, 388 (0.5%) had experienced at least one admission to hospital for hypoglycaemia. In relation to the index date, the closest hypoglycaemic event occurred within 1 year in around 30% of subjects and within 2 years in around 50% (electronic supplementary material [ESM] Fig. 1).

Subjects who experienced a severe hypoglycaemic event were older, more frequently of white ethnicity and more deprived, with a lower BMI, a lower eGFR and worse glycaemic control (Table 1). Further differences between the two groups were observed for medications and comorbidities: insulin therapy was more frequent in people with a severe hypoglycaemic episode (absolute difference 28.7%), as well as a medical history of cancer (absolute difference 2.7%), myocardial infarction (5.7%), peripheral artery disease (9.1%), atrial fibrillation (12.9%), heart failure (14.5%) or stroke (15.1%). Differences in smoking habit, systolic blood pressure and total cholesterol between the two groups were marginal.

**Table 1:** Baseline characteristics of the cohort and number of events

Variable	Hospital admission for severe hypoglycaemia	
	No *N* = 74,222	Yes *N* = 388
Age (years)	67.7 (58.4–76.5)	73.9 (60.3–81.8)
Sex		
Men	40,677 (54.8%)	195 (50.3%)
Women	33,545 (45.2%)	193 (49.7%)
Ethnicity		
White	66,606 (89.7%)	362 (93.3%)
Non-white	7616 (10.3%)	26 (6.7%)
Townsend score (fifths)		
1 (least deprived)	15,245 (20.5%)	44 (11.3%)
2	16,325 (22.0%)	75 (19.3%)
3	15,454 (20.8%)	86 (22.2%)
4	16,067 (21.6%)	98 (25.3%)
5 (most deprived)	11,131 (15.0%)	85 (21.9%)
Smoking status		
Current	10,695 (14.4%)	66 (17.0%)
Former	29,043 (39.1%)	137 (35.3%)
Never	34,484 (46.5%)	185 (47.7%)
Alcohol consumption		
Current	52,435 (70.6%)	229 (59.0%)
Former	4223 (5.7%)	34 (8.8%)
Never	17,564 (23.7%)	125 (32.2%)
Systolic BP (mmHg)	135 (125–143)	132 (122–142)
Total cholesterol (mmol/l)	4.2 (3.6–4.9)	4.0 (3.4–4.8)
HbA_1c_ (mmol/mol)	52 (45–61)	58 (46–73)
HbA_1c_ (%)	6.9 (6.3–7.7)	7.5 (6.4–8.8)
BMI (kg/m^2^)	30.1 (26.6–34.5)	28.3 (24.1–33.1)
eGFR (ml min^−1^ 1.73 m^−2^)	77 (61–91)	63 (42–85)
Medications		
Metformin	36,757 (49.5%)	107 (27.6%)
Sulfonylurea	15,527 (20.9%)	76 (19.6%)
Insulin	4904 (6.6%)	137 (35.3%)
Other glucose-lowering	8019 (10.8%)	17 (4.4%)
Statin	49,077 (66.1%)	206 (53.1%)
Antiplatelet drugs	12,165 (16.4%)	69 (17.8%)
Comorbidities		
Myocardial infarction	6679 (9.0%)	57 (14.7%)
Stroke	7338 (9.9%)	97 (25.0%)
Peripheral artery disease	2826 (3.8%)	50 (12.9%)
Heart failure	4539 (6.1%)	80 (20.6%)
Atrial fibrillation	7631 (10.3%)	90 (23.2%)
Cancer	10,017 (13.5%)	63 (16.2%)
Mortality		
All-cause		
Event	18,539 (25.0%)	236 (60.8%)
Rate (per 1000 person-years)	40 (39, 41)	132 (117, 150)
CVD		
Event	5400 (7.3%)	68 (17.5%)
Rate (per 1000 person-years)	12 (11, 12)	38 (30, 48)
Cancer		
Event	4929 (6.6%)	27 (7.0%)
Rate (per 1000 person-years)	11 (10, 11)	15 (10, 22)
Other causes		
Event	8210 (11.1%)	141 (36.3%)
Rate (per 1000 person-years)	18 (17, 18)	79 (67, 93)

Numbers are reported as median (interquartile range) or number (percentage), except for rate (95% CI)

### Cause-specific mortality

During a median follow-up of 7.1 years (465,576 person-years), 18,775 deaths occurred: 236 (60.8%) in subjects with previous severe hypoglycaemia and 18,539 (25.0%) in those without, corresponding to a mean mortality rate of 132 (95% CI 117, 150) and 40 (95% CI 39, 41) per 1000 person-years, respectively. Overall, non-cardiovascular and non-cancer causes accounted for the majority of deaths (44.5%), followed by cardiovascular (29.1%) and cancer (26.4%) causes (Table 1). There were 69 other causes of death in subjects with severe hypoglycaemia, of which five causes accounted for one-third of the total deaths: ‘unspecified dementia’ (ICD-10 F03; 11.3%); ‘unspecified pneumonia’ and ‘unspecified bronchopneumonia’ (J18.9 and J18.0, respectively; both 6.4%); ‘vascular dementia’ (F01.9; 5.0%); and ‘chronic obstructive pulmonary disease with acute lower respiratory infection’ (J44.0; 4.3%) (ESM Fig. 2). For the same causes of death, the corresponding proportions in subjects without severe hypoglycaemia were very similar, with the largest difference observed for ‘chronic obstructive pulmonary disease with acute lower respiratory infection’ (J44.0), which accounted in these subjects for 5.6% of other deaths. Among all causes of death, the largest crude difference was observed for renal complications, which were 3% more frequent in subjects with severe hypoglycaemia. Comparing subjects with and without hypoglycaemia, the unadjusted absolute difference in the mean mortality rate was 26 per 1000 person-years higher for cardiovascular death, 4 for cancer death and 61 for other causes of death. These estimates corresponded to absolute risk differences at the end of follow-up of 10.2%, 0.4% and 25.2%, respectively (Table 1).

**Fig. 2:**
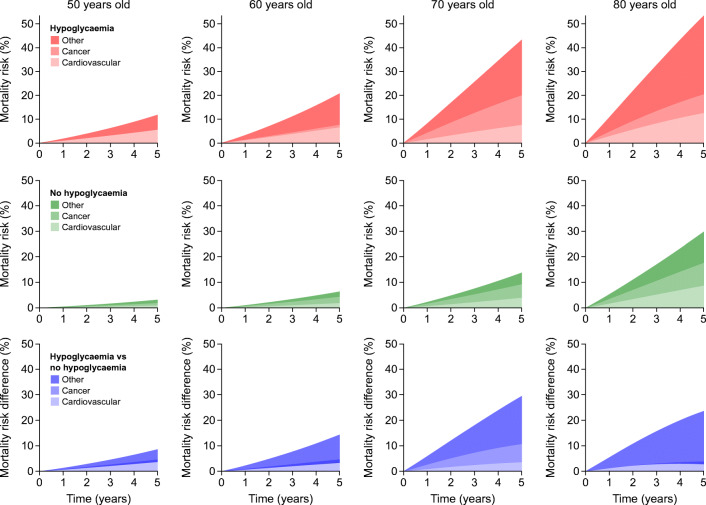
Cause-specific mortality over 5 years. Adjusted probabilities of cause-specific death are shown for 5 years of follow-up for different ages, in subjects with (red) and without (green) hypoglycaemia; the differences in the probabilities are shown in blue. In each panel, the transparency of the colour indicates the cause of death: from bottom to top, most transparent, cardiovascular; middle transparency, cancer; least transparent, other causes. As the probabilities are stacked, the overall area indicates the probability of all-cause mortality

In subjects with severe hypoglycaemia, mortality increased progressively over 5 years for all ages, with differences among causes of death (Fig. 2). For a 50-year-old individual, the 5 year CVD mortality risk was 5.6% and increased to 6.6%, 7.6% and 12.6% at 60, 70 and 80 years old, respectively. The pattern of cancer mortality indicated a roughly constant risk over 5 years at the ages of both 50 and 60 years (mortality <1.1%); a 5 year mortality risk of 12.4% at 70 years old; and 7.9% at 80 years old. Lastly, for other causes of death, the risk steadily increased over time at all ages, resulting in 6.2%, 13.1%, 23.3% and 33.0% at 50, 60, 70 and 80 years old, respectively.

In a 50-year-old individual without hypoglycaemia, the 5 year CVD mortality risk was 1.0% and increased to 1.9%, 4.0% and 8.8% at 60, 70 and 80 years old, respectively (Fig. 2). The 5 year risk of cancer death was 0.9% at 50 years old, 2.5% at 60 years old, 5.3% at 70 years old and 8.9% at 80 years old. Corresponding estimates for other causes of death were 1.3%, 2.0%, 4.5% and 12.1%.

**Fig. 3:**
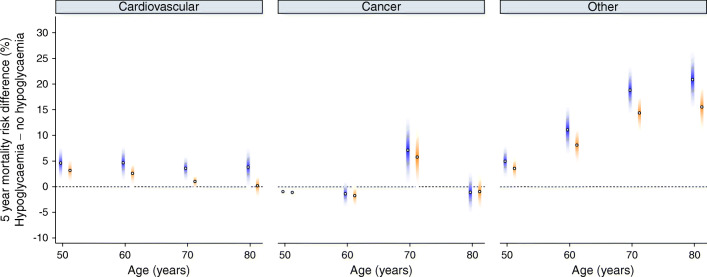
Effects of confounders on 5 year cause-specific and all-cause mortality differences. Cause-specific 5 year mortality differences across ages are shown for a model including sex, ethnicity, systolic blood pressure, total cholesterol, HbA_1c_, BMI, eGFR, smoking status, alcohol consumption, Townsend score and a non-linear interaction between age and hypoglycaemia status (model 1, blue). Corresponding estimates, upon further adjustment for glucose-lowering medications (metformin, sulfonylurea, insulin, others), comorbidities (myocardial infarction, stroke, peripheral artery disease, heart failure, atrial fibrillation, cancer) and cardioprotective medications (statin and antiplatelet drugs), are shown in orange (model 2). Shaded colours indicate 95% CIs

Differences in the mortality risk comparing subjects with and without severe hypoglycaemia were of moderate magnitude for CVD death, with 5 year absolute differences of 4.6% (95% CI 1.2, 8.0), 4.7% (1.0, 8.3), 3.6% (0.9, 6.3) and 3.8% (−0.1, 7.8) at 50, 60, 70 and 80 years old, respectively (Fig. 3; ESM Table 1). Corresponding estimates were −0.9% (−1.1, −0.8; indicating a greater risk in subjects without hypoglycaemia), −1.4% (−4.1, 1.4), 7.1% (0.4, 13.8) and −1.1% (−5.3, 3.1) for cancer mortality; and 5.0% (1.6, 8.3), 11.1% (6.1, 16.1), 18.8% (14.0, 23.7) and 20.9% (15.2, 26.6) for other causes.

None of the variables added in model 2 had missing data. From the same sample of 74,610 with no missing data, the complete-case analyses accounting also for medications and comorbidities showed increased mortality rates associated with sulfonylurea (cause-specific hazard ratio 1.13 [95% CI 1.05, 1.20] for cardiovascular; 1.06 [0.99, 1.14] for cancer; and 1.20 [1.14, 1.27] for other causes of death) and insulin (1.42 [95% CI 1.29, 1.56]; 1.21 [1.08, 1.36]; and 1.35 [1.24, 1.48], respectively) treatment, and smaller differences in the risk of cause-specific death compared with the results of the main analysis (Fig. 3; ESM Table 1), particularly in older subjects and for other causes of death.

### Sensitivity analysis and multiple imputations

In a sensitivity analysis including time since admission for severe hypoglycaemia in the main analysis model, the risk estimates were not materially changed (ESM Table 2). Results obtained in the sample of 86,199 subjects following imputation of missing data were overlapping with those obtained in model 2 (ESM Table 1).

## Discussion

In this study of primary care subjects with type 2 diabetes, we observed a high risk of death in people with a prior admission to hospital for a severe hypoglycaemic episode: in a 60-year-old individual, the 5 year risk of death was around 7% for CVD causes, 1% for cancer causes, and 13% for non-CVD and non-cancer causes, resulting in an overall risk of around 21%. The corresponding risk in a person without severe hypoglycaemia was 2%, 3% and 2%, resulting in an absolute mortality risk difference associated with severe hypoglycaemia of 14%. Accounting for missing data and for a large set of confounders strongly related to the risk of death, the estimate was more conservative and indicated an absolute risk difference of 11%.

Hypoglycaemia in individuals with diabetes has long been recognised as an acute, frequent and, in most cases, reversible complication of intensive glucose control. The scientific interest around hypoglycaemia reignited a decade ago, after the publication of randomised controlled trials demonstrating a neutral or higher risk of death in individuals with type 2 diabetes randomised to an intensive glucose control. Hypoglycaemia was deemed a potential reason, with severe, repeated hypoglycaemic episodes not only thought to pose a risk for acute symptoms and complications but also to predispose an individual to long-term CVD events through direct (i.e. ECG abnormalities) and indirect (i.e. platelet abnormalities) mechanisms [2]. The clinical trade-off would therefore be between long-term complications of hyperglycaemia and short- and long-term complications of hypoglycaemia. As a result, there has been increasing emphasis on inappropriate intensive glucose control in individuals at a higher risk of hypoglycaemia (overtreatment) [11].

In light of the rising prevalence of severe hypoglycaemia in multimorbid people with type 2 diabetes [12–14], clarifying whether hypoglycaemia is causally related to an increased risk of CVD remains important from both a patient and a public health perspective. Most of the available evidence from epidemiological studies, in different geographical regions and from heterogeneous groups of patients with type 2 diabetes, has shown a positive association between severe or non-severe hypoglycaemia and risk of CVD events or death [2, 3, 5, 6, 15–27]; these observations, however, have been inconsistent [25, 28]. In the attempt to quantify causality, previous studies adjusted for several potential confounders and explored associations with less-plausible outcomes (i.e. negative control outcomes). Virtually all, however, have estimated the risk of complications in terms of hazard ratio, which is difficult to interpret and lacks information on the absolute disease burden. In fact, in a population at a low absolute risk of an event, an intervention has smaller public health implications than in a population at a high absolute risk.

To our knowledge, few studies have specifically quantified the absolute risk of outcomes associated with severe hypoglycaemia in individuals with type 2 diabetes. Zhao et al designed a matched cohort study of 1522 participants with type 2 diabetes (mean age, 63 years) without cardiovascular and microvascular diseases using the Veterans Health Administration electronic health records. In 761 participants reporting a previous episode of any hypoglycaemia, the 3 year risks of fatal and non-fatal CVD events and mortality were 34% and 9%, respectively, while the 5 year risk of CVD events was about 43%; corresponding estimates at 3 years in the 761 participants without hypoglycaemia were 22% for CVD events and 7% for mortality [18]. By applying the same study design, Jensen et al analysed data from 10,130 individuals with type 2 diabetes (mean age, 74 years): the 5 year risk of death was 62% in those admitted to hospital for severe hypoglycaemia (5605 individuals) and 37% in the matched participants without severe hypoglycaemia [29]. In 1209 participants with type 2 diabetes (mean age, 64 years) enrolled in the Atherosclerosis Risk in Communities cohort study, Lee et al investigated the risks of CVD events and all-cause death associated with hypoglycaemia, defined as an episode resulting in an ambulance call, emergency department visit or hospitalisation. In the 195 participants with hypoglycaemia, the unadjusted risk 3 years after the hypoglycaemic episode was about 11% for coronary heart disease and 28% for mortality [20]. Of note, in this study hypoglycaemia was associated with an increased relative risk of CVD mortality and cancer mortality but not non-CVD and non-cancer mortality. Similar to this study, other authors have reported the cumulative risk of CVD events or death without accounting for the heterogeneous clinical characteristics between patients with and without hypoglycaemia, potentially resulting in an overestimation of the difference [19, 21, 30]. Comparisons between these studies and our results are difficult because of the different definitions of hypoglycaemia, outcomes, population, design and analytical approach. In particular, in estimating the absolute risk, previous studies did not account for the competing nature of the events and the majority did not adjust for key confounders (i.e. age).

Our results are, however, in line with previous studies showing an increased risk for outcomes that may be considered negative controls [2, 6, 29]. In both patients with and patients without hypoglycaemia, the most common cause of death in our study was not a CVD event, as the combination of cancer and other causes accounted for around 71% of all deaths. Furthermore, the 5 year risk difference between hypoglycaemia and non-hypoglycaemia, adjusted for the heterogeneous clinical characteristics between the two groups, was higher for other causes of death (11%) than for CVD causes (5%). Among the other causes of death, the combination of ‘unspecified’, ‘vascular’ and ‘Alzheimer’s’ dementia accounted for 19% of all other causes of death in subjects with severe hypoglycaemia. We recognise that dementia may not be considered a negative control outcome as several vascular and nonvascular mechanisms could link hypoglycaemia to dementia [31]; yet, the same combination of the three causes accounted for a very similar proportion also in subjects without hypoglycaemia (i.e. 20%). As these two estimates are unadjusted and the epidemiological evidence in this area is contrasting [32–35], further studies should be conducted, ideally with a detailed phenotypical assessment of dementia to minimise coding misspecification.

In contrast with dementia, to our knowledge, no established mechanisms link hypoglycaemia to bronchopulmonary diseases, although they accounted for significant and very similar proportions of other causes of death in both subjects with and subjects without severe hypoglycaemia. The possibility of non-causality is further supported by the reduction in the estimated differences upon the inclusion of further potential confounders, suggesting the presence of residual confounding. This is in line with previous observational studies indicating that hypoglycaemia and CVD share several risk factors [13], as well as with the bidirectional nature of the association between hypoglycaemia and CVD [24, 36]. Together with the available evidence, our results would therefore strongly point towards a non-causal association between hypoglycaemia and long-term CVD complications in type 2 diabetes. However, regardless of the nature of the association, from a prognostic perspective our study identifies a group of patients with type 2 diabetes at a very high risk of death.

Our study extends previous research on the relationship between hypoglycaemia and risk of death. We defined a large and contemporary cohort of primary care subjects with type 2 diabetes, thus enhancing precision and generalisability of the results. We also focused on the absolute risk and accounted for the competing causes of death. As age is the strongest risk factor of death, we estimated the absolute risks and their differences for different ages; this enables age-specific prognostic comparisons with other risk factors or diseases (i.e. cancer).

This study also has some limitations. We defined hypoglycaemia as an episode of hospitalisation reporting hypoglycaemia as the underlying cause. From a clinical perspective, hypoglycaemia itself may not be the sole reason for admission; the decision to admit could be related to a patient’s clinical complexity, which is associated with an increased risk of death. Our results confirmed this hypothesis; we adjusted for several potential confounders to minimise confounding bias, yet our results would suggest that a residual confounding is still possible. We considered only admission to hospital because both severe hypoglycaemic episodes without hospitalisation and non-severe episodes, available only from CPRD, represent a more heterogeneous exposure. The definition used in this study and the necessity to link detailed clinical information to hospital data also resulted in a relatively small number of subjects with severe hypoglycaemia. Although the use of glucose-lowering medications associated with a lower risk or no risk of hypoglycaemia is increasing [37–39], the available evidence shows that the number of patients admitted to hospital for severe hypoglycaemia as the underlying cause was around 79,000 in England during the decade 2005–2014 [12]; this figure further highlights the public health relevance of our findings. Moreover, using this definition for the exposure may have resulted in an overestimation of the absolute risk of mortality in people admitted to hospital for a severe hypoglycaemic episode and, in turn, in a larger difference in the mortality risk compared with those without severe hypoglycaemia. In fact, severe hypoglycaemic episodes not requiring hospitalisation likely occur in individuals with a lower risk of complications and death. Whether the possible overestimation similarly applies to different causes of death, however, is difficult to envisage.

We used a prevalent cohort design and estimated the age-specific absolute risk of different causes of death in order to investigate the biological plausibility of the association between hypoglycaemia and cause-specific death; of note, when time since the last hypoglycaemic episode was included in the analysis, estimates did not materially change. An incident cohort design, whereby the occurrence of hypoglycaemia is the starting time of follow-up, is more relevant when the goal is to examine temporality, which is another criterion to judge causality. The temporal relationship of hypoglycaemia with CVD events and all-cause mortality has been variably investigated and reported, with some studies showing a constant, and others a reduced, relative risk over time [24, 27]. In this respect, it is also worth mentioning that we explored the long-term risk of mortality associated with hypoglycaemia; however, in acute or sub-acute clinical settings, hypoglycaemia may be the cause (i.e. injuries) or may contribute to a worse prognosis (i.e. infection) for ‘other’ causes of death. Further epidemiological studies should be specifically designed to explore temporality, accounting for the competing nature of the outcomes and the possible varying magnitudes of the associations between hypoglycaemia and each outcome over time. Lastly, similar to other large, observational studies using electronic health records, the risk of miscoding for both the exposure and the outcome cannot be excluded.

Severe hypoglycaemia resulting in hospitalisation is an event that carries a high long-term risk of death. As most of the causes of death in our study were not related to CVD, for which several pathophysiological mechanisms have been proposed, and a large set of potential confounders were accounted for, our results are highly suggestive for a non-causal relationship between severe hypoglycaemia and long-term CVD complications. Rather, severe hypoglycaemia is very likely a marker of frailty, which is causally associated with a higher risk of death. Along with the clinical principle of reducing glucose without causing hypoglycaemia to avoid short-term complications and preserve the quality of life in patients with diabetes [40], we underline that hospitalisation for hypoglycaemia identifies clinically complex phenotypes of type 2 diabetes: in these patients, further research should be conducted to identify the optimal strategies to reduce the risk of death.

## Electronic supplementary material

ESM (PDF 952 kb)

## Data Availability

Statistical codes and all codes used to identify the population, main exposure, covariates and outcomes are available on GitHub (frazac82). CPRD data governance does not allow distributing patient data to other parties. Researchers may apply for data access at http://www.CPRD.com/. The study is registered with CPRD study protocol 18_157R2.

## Abbreviations

ACCORD: Action to Control Cardiovascular Risk in Diabetes
CPRD: Clinical Practice Research Datalink
HES: Hospital Episode Statistics
ICD: International Classification of Diseases
ONS: Office for National Statistics
