# Supplementary material for: Severe hypoglycaemia and absolute risk of cause-specific mortality in individuals with type 2 diabetes: a UK primary care observational study
Source: Diabetologia. 2020 Jul 30;63(10):2129–39. doi: 10.1007/s00125-020-05223-3 (PMC7476909; doi:10.1007/s00125-020-05223-3)
Supplement: Supplementary file 1 — (PDF 952 kb) [file 125_2020_5223_MOESM1_ESM.pdf]

ELECTRONIC SUPPLEMENTARY MATERIAL

Severe hypoglycaemia and absolute risk of cause-specific mortality in individuals with type 2 diabetes: A UK primary care observational study

Francesco Zaccardi, Suping Ling, Claire Lawson, Melanie J Davies, Kamlesh Khunti

Contents

ESM Table 1: Multiple imputation and complete-case results 2

ESM Table 2: Time since hypoglycaemia and risk of outcomes 3

ESM Figure 1: Temporal pattern of severe hypoglycaemia, main analysis 4

ESM Figure 2: Other causes of death, main analysis 5

ESM Table 1: Multiple imputation and complete-case results

Age	Model	Cardiovascular	Cancer	Other
50	Complete case – model 1	4.6 (1.2, 8.0)	-0.9 (-1.1, -0.8)	5.0 (1.6, 8.3)
	Complete case – model 2	3.2 (0.6, 5.7)	-1.1 (-1.2, -0.9)	3.6 (0.9, 6.2)
	Multiple imputation	3.6 (0.9, 6.2)	-1.0 (-1.1, -0.9)	4.0 (1.3, 6.7)
60	Complete case – model 1	4.7 (1.1, 8.3)	-1.4 (-4.1, 1.4)	11.1 (6.1, 16.1)
	Complete case – model 2	2.6 (0.0, 5.2)	-1.7 (-4.2, 0.7)	8.1 (4.2, 12.0)
	Multiple imputation	2.9 (0.3, 5.4)	-1.4 (-3.9, 1.1)	9.2 (5.2, 13.3)
70	Complete case – model 1	3.6 (0.9, 6.3)	7.1 (0.4, 13.8)	18.8 (14.0, 23.7)
	Complete case – model 2	1.0 (-0.8, 2.9)	5.8 (-0.1, 11.7)	14.4 (10.3, 18.4)
	Multiple imputation	0.7 (-0.9, 2.4)	5.0 (-0.3, 10.3)	14.9 (11.0, 18.7)
80	Complete case – model 1	3.8 (-0.1, 7.8)	-1.1 (-5.3, 3.1)	20.9 (15.2, 26.6)
	Complete case – model 2	0.2 (-2.4, 2.8)	-0.9 (-4.7, 2.8)	15.5 (10.6, 20.4)
	Multiple imputation	-0.7 (-2.9, 1.6)	-0.9 (-4.3, 2.6)	15.1 (10.6, 19.6)

Shown are 5-year mortality differences (%) with 95% CI comparing hypoglycaemia vs no hypoglycaemia.

In the main analysis (**Complete case – model 1**), estimates were adjusted for age, sex, ethnicity, systolic blood pressure, total cholesterol, HbA1c, BMI, eGFR, smoking status, alcohol consumption, Townsend score.

In **complete case – model 2** and **multiple imputation**, estimates were further adjusted for glucose-lowering medications (metformin, sulphonylurea, insulin, others), comorbidities (myocardial infarction, stroke, peripheral artery disease, heart failure, atrial fibrillation, cancer), and cardioprotective medications (statin and antiplatelet drugs).

ESM Table 2: Time since hypoglycaemia and risk of outcomes

Model	Cause-specific hazard ratio (95% confidence interval)		
	**Cardiovascular**	**Cancer**	**Other**
Complete case – model 1	2.93 (2.13, 4.02)	0.07 (0.00, 5.51)	4.76 (3.79, 5.97)
Complete case – model 1 + time since hypoglycaemia	3.24 (2.15, 4.88)	0.08 (0.00, 6.35)	4.83 (3.59, 6.49)

Hazard ratios of cause-specific death, comparing admission for severe hypoglycaemia vs no-admission for severe hypoglycaemia, were estimated in the main analysis (**Complete case – model 1**, adjusted for age, sex, ethnicity, systolic blood pressure, total cholesterol, HbA1c, BMI, eGFR, smoking status, alcohol consumption, Townsend score) and upon further inclusion of time since last (i.e. closest to index date) hypoglycaemic episode. This variable was set to zero for subjects who did not experience severe hypoglycaemia (unexposed).

ESM Figure 1: Temporal pattern of severe hypoglycaemia, main analysis

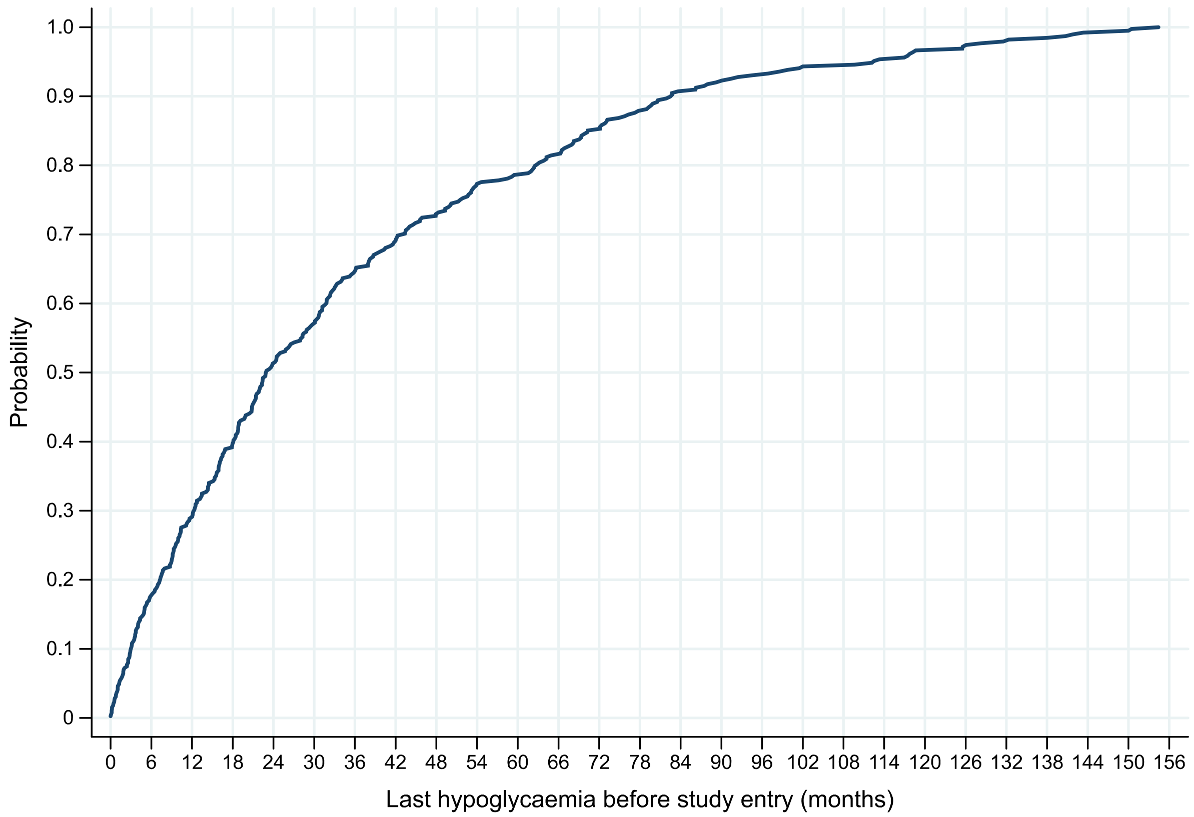

ESM Figure 2: Other causes of death, main analysis

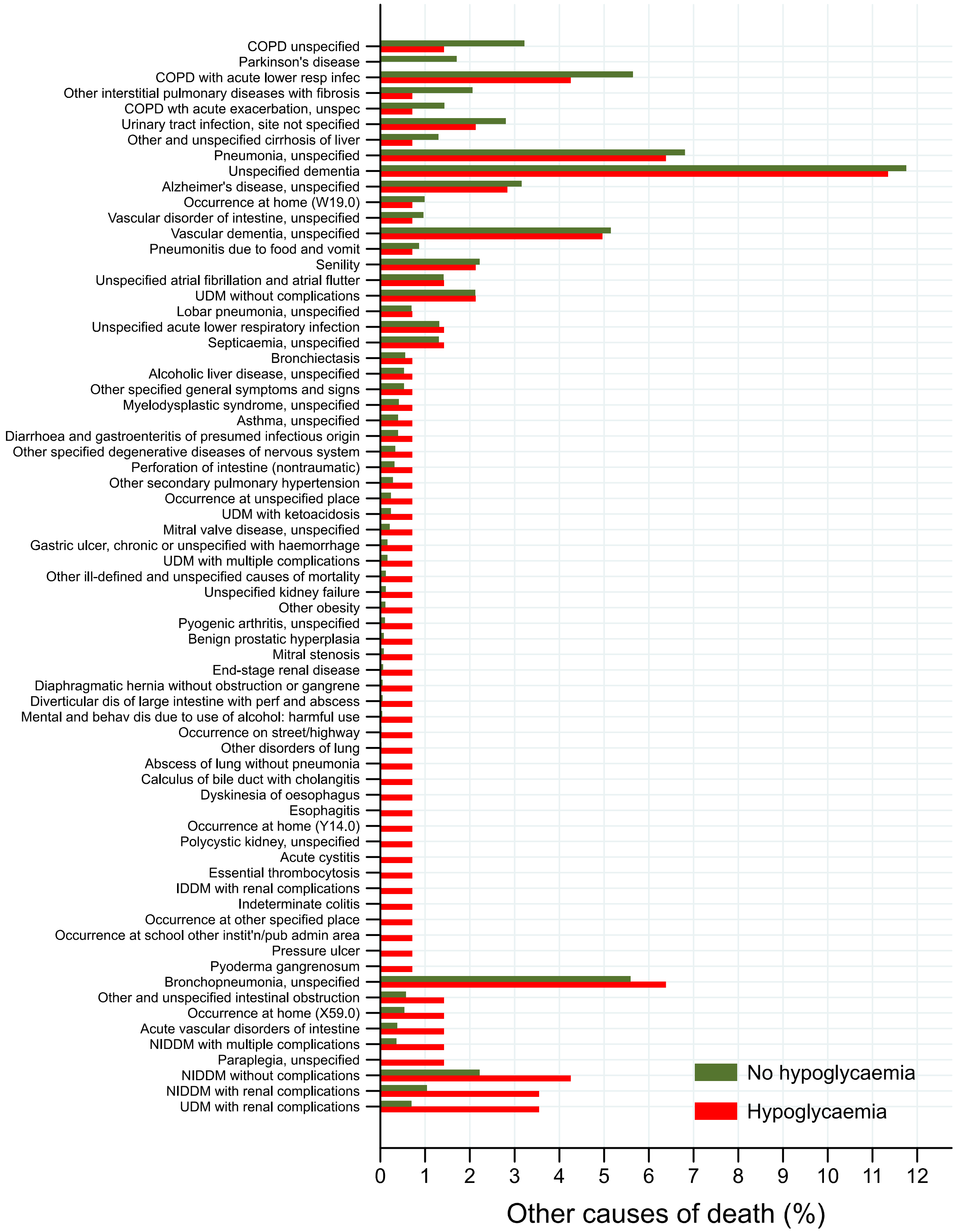

Diseases are sorted in ascending order of the difference between the proportions in subjects with (red) vs without (green) severe hypoglycaemia (i.e., more frequent in subjects with hypoglycaemia at bottom).

Shown are the proportion (%) of each other cause of death out of all other causes of death; all other causes of death in subjects with severe hypoglycaemia are reported (n=69), corresponding to 73.9% of other causes of death in subjects without hypoglycaemia.

COPD: Chronic Obstructive Pulmonary Disease; **IDDM**: Insulin-Dependent Diabetes Mellitus; **NIDDM**: Non-Insulin Dependent Diabetes Mellitus; **UDM**: Unspecified Diabetes Mellitus.
